# Subthalamic nucleus deep brain stimulation in elderly patients – analysis of outcome and complications

**DOI:** 10.1186/1471-2377-7-7

**Published:** 2007-03-16

**Authors:** Jan Vesper, Susanne Haak, Christoph Ostertag, Guido Nikkhah

**Affiliations:** 1 Department of Stereotactic Neurosurgery, Neurocenter, University of Freiburg, Germany

## Abstract

**Background:**

There is an ongoing discussion about age limits for deep brain stimulation (DBS). Current indications for DBS are tremor-dominant disorders, Parkinson's disease, and dystonia. Electrode implantation for DBS with analgesia and sedation makes surgery more comfortable, especially for elderly patients. However, the value of DBS in terms of benefit-risk ratio in this patient population is still uncertain.

**Methods:**

Bilateral electrode implantation into the subthalamic nucleus (STN) was performed in a total of 73 patients suffering from Parkinson's disease. Patients were analyzed retrospectively. For this study they were divided into two age groups: group I (age <65 years, n = 37) and group II (age ≥ 65 years, n = 36). Examinations were performed preoperatively and at 6-month follow-up intervals for 24 months postoperatively. Age, UPDRS motor score (part III) on/off, Hoehn & Yahr score, Activity of Daily Living (ADL), L-dopa medication, and complications were determined.

**Results:**

Significant differences were found in overall performance determined as ADL scores (group I: 48/71 points, group II: 41/62 points [preoperatively/6-month postoperatively]) and in the rate of complications (group I: 4 transient psychosis, 4 infections in a total of 8 patients, group II: 2 deaths [unrelated to surgery], 1 intracerebral hemorrhage, 7 transient psychosis, 3 infections, 2 pneumonia in a total of 13 patients), (p < 0.05). Interestingly, changes in UPDRS scores, Hoehn & Yahr scores, and L-dopa medication were not statistically different between the two groups.

**Conclusion:**

DBS of the STN is clinically as effective in elderly patients as it is in younger ones. However, a more careful selection and follow-up of the elderly patients are required because elderly patients have a higher risk of surgery-related complications and a higher morbidity rate.

## Background

Chronic high-frequency deep brain stimulation (DBS) has evolved into an established therapeutic approach for treating patients with extrapyramidal movement disorders, in particular patients with Parkinson's disease. DBS effectively suppresses tremor and rigor as well as akinesia and dyskinesia [1-14].

Numerous studies confirm the safety and efficacy of DBS. Various targets have been used for suppressing specific symptoms. The subthalamic nucleus (STN) is the preferred surgical target for DBS in Parkinson's disease [4,12,15-20].

Data on complications of the intervention are inconsistent and cannot be compared. Adverse events associated with STN stimulation are quite common with a high incidence of mental changes though these are typically transient in nature [21-30].

Only limited data are available on the long-term clinical course of patients after DBS surgery [31-34]. Because of this lack of data, it has not been possible so far to definitively answer questions regarding an age limit for DBS, a possible loss of effectiveness over time, or potential neuroprotective effects of DBS [35-37]. The study presented here aimed at determining whether STN DBS is as effective in elderly patients as it is in younger ones and whether there are any differences in the long-term outcome of treatment between older and younger patients.

We therefore performed a retrospective analysis of all patients who underwent electrode implantation for DBS at two centers and were followed up for two years. Since there were no significant differences in patient age and performance, in the complication rate and in the results of the DBS, we summarized the two centers. The patients were divided into two age groups to compare results of DBS in elderly individuals (> 65 years) with those in younger patients (< 65 years). The clinical outcome and complications of the operation and DBS were compared between the two age groups.

## Methods

A total of 73 patients were included in the statistical analysis, 51 patients operated on at center A and 22 patients operated on at center B. The patients of both centers had a mean history of Parkinson's disease at the time of surgery of 14.6 years.

The patients were divided into two groups for analyzing effects of age on outcome: Group I included 37 patients aged up to 65 and group II included 36 patients aged 65 or above. The cut off was chosen because 65 year limit is also known for therapy decisions e.g. in neurooncological diseases, due to increased comorbidity in elderly patients [38]. The age range of the total study population operated on was 44 to 76 years (mean 64.1 ± 7.2 years). There was no significant difference in the age distribution between the two participating centers. For evaluation the patients were summarized.

The patients were assessed using the Unified Parkinson's Disease Rating Scale (UPDRS), the Activity of Daily Living scale (ADL, Schwab and England), and the Hoehn & Yahr scale. In addition, changes in medication as well as complications and adverse events associated with surgery were recorded. Complications were defined as all events that considerably prolonged a patient's hospital stay or required treatment because they impaired quality of life. Adverse events were defined as stimulation-dependent problems responding to changes in stimulation parameters.

UPDRS data at 24-month follow-up were available from 42 of 51 patients in center A and from 16 of 22 patients in center B. Stimulation parameters, Hoehn & Yahr scores, ADL scores, and data on complications and adverse events were available from all 73 patients.

### Operative procedure

Electrodes were stereotactially implanted into the STN on both sides in all patients. The intended target coordinates relative to the mid-commissural point (MC) were as follows: x = ± 11 mm, y = -2 mm, and z = -2 mm. The target coordinates were determined on the basis of ventriculography, computerized tomography, and intraoperative image fusion with a three-dimensional MRI dataset. Surgery was initially performed under local anesthesia, later with analgesia and sedation (propofol and alfentanil). Since no revisions or removal of the electrodes under external stimulation became necessary in any of the patients, we dispensed with external stimulation in subsequent implantations since 1999. The pulse generator (bilateral Itrel^® ^II until 1998; since 1999 Kinetra™, Medtronic Inc., Minneapolis, MN, USA,) was implanted under general anesthesia.

### Statistical evaluation

Statistical testing of the results was done by multivariate analysis using the Wilcoxon signed rank test and the one-way ANOVA rank sum test (Sigmastat 1.0, Jandel Scientific Inc, Chicago, IL, USA).

## Results

**Figure 1:**
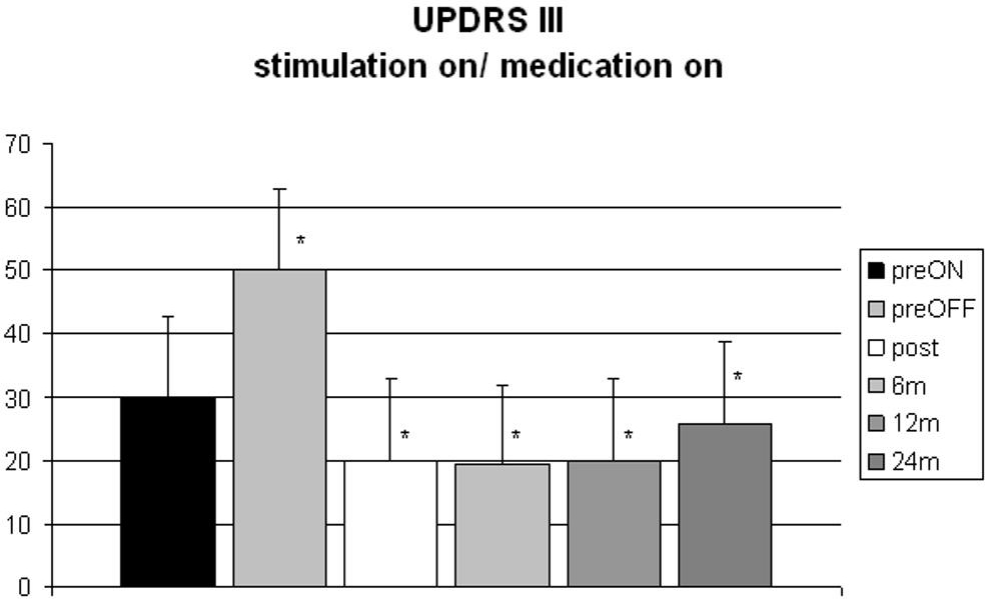
UPDRS motor scores (part III) in all patients before surgery (with/without medication) and at postoperative follow-up after 6, 12, and 24 months (preoperative medication on – preON, preoperative medication off: preOFF, postoperative stimulation on and medication on).

Follow-up data after 24 months were available from 58 patients. UPDRS Part III scores showed a persistent improvement of motor function (Fig. 1). No differences between the two age groups and the two centers were seen for the different conditions investigated (preoperatively on medication/off medication and postoperatively on stimulation/on medication, off stimulation/off medication, on stimulation/off medication, and off stimulation/on medication) (Tab. 1). A lessening of the stimulation effect or progression of the underlying disease was not observed during the study period.

**Figure 2:**
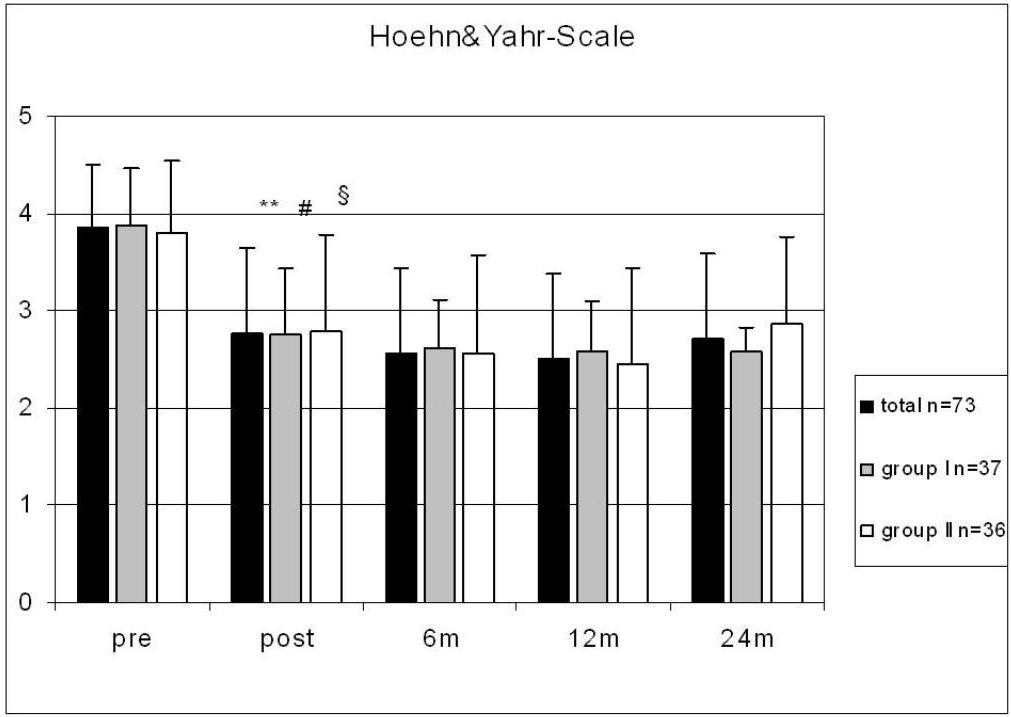
Hoehn and Yahr scores in all patients as well as in groups I and II preoperatively (with/without medication) and at follow-up after 6, 12, and 24 months; significant differences between pre- and postoperative scores in all groups (**, ^#^, ^§ ^p < 0.05).

The Hoehn & Yahr scores showed a significant improvement for the "medication off" and "stimulation on" state (mean total score: 3.9 preoperatively versus 2.8* postoperatively, 2.6 at 6-month follow-up, and 2.7 at 24-month follow-up, *p < 0.05, significant difference in scores before and after stimulator implantation). The difference was significant for the study population as a whole and for the two age groups (p < 0.05). The differences between two age groups were not significant (Fig. 2).

**Figure 3:**
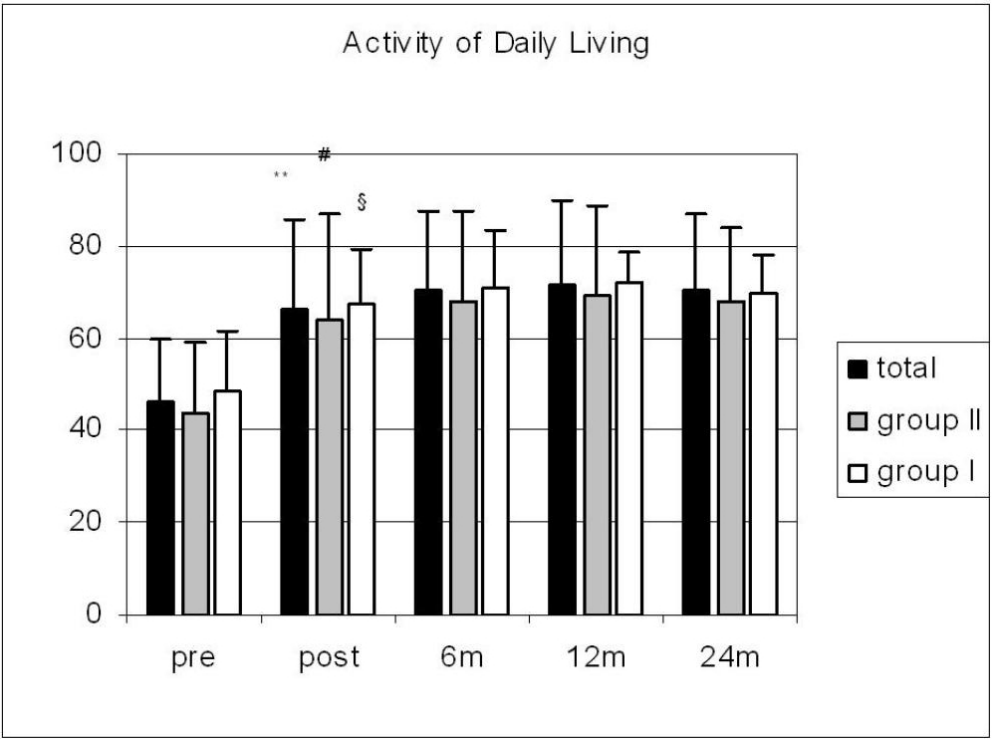
Activity of Daily Living scores in all patients as well as in groups I and II preoperatively (with/without medication) and at the different postoperative follow-up times; significant differences between pre- and postoperative scores in all groups (**, ^#^, ^§ ^p < 0.05).

**Figure 4:**
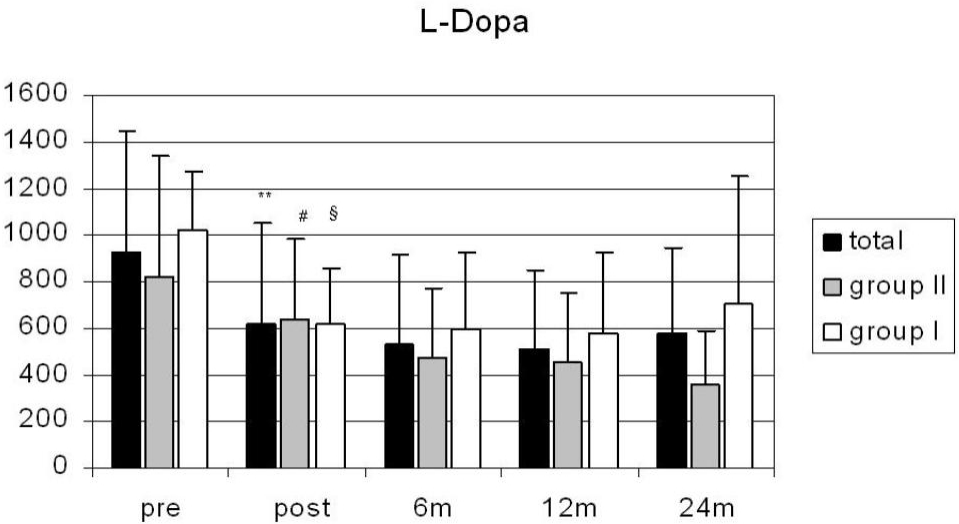
L-dopa equivalents (mg) in all patients as well as in groups I and II preoperatively and at the different postoperative follow-up times; significant differences between pre- and postoperative state in all groups (**, ^#^, ^§ ^p < 0.01), 24 m group I: ns.

The patients' quality of life also improved significantly. The ADL scores improved homogeneously in both groups (Fig. 3). L-Dopa equivalent medication could be reduced by a mean of 45% during the first 12 months after electrode implantation (p < 0.01). The dose reduction was significant for the total study population (n = 73) and for the two age groups after 6 and 12 months (p < 0.01) (Fig. 4).

Stimulation parameters were constant during the first twelve months after electrode implantation. For tremordominant Parkinson's disease higher frequencies were used to suppress tremor symptoms. (Table 3)

### Complications and side effects

A total of 27 complications, defined as unexpected events prolonging a patient's hospital stay, occurred in 20 patients (Tab. 2). Severe complications were rare. One patient suffered from symptomatic hemorrhage. The transient hemiparesis resulted in a complete recovery after 6 months. The two age groups differed with regard to the death rate during the follow-up period and the incidence of infections. One patient in group I committed suicide after one year. Another 6 patients died from causes unrelated to the operation during the follow-up period. Infections were more common in group II (n = 1 vs. n = 4, p < 0.05). All but one infection occurred at the site of the stimulator pouch. The remaining infection occurred at the extension connector site. In all these cases the whole stimulation system was explanted. Patient underwent systemic specific antibiotic therapy for two weeks and oral therapy for another 4 weeks. After 3 months reimplantation took place. In all patients of this series the previous stimulation effect could have been maintained.

Transient psychic deterioration frequently occurred in both groups of patients. Although side effects of stimulation were frequent, especially stimulation dependent dysarthria, there was no permanent neurological morbidity in this study. By changing the stimulation parameters or the active contacts, the effect of DBS was not diminished.

## Discussion

Deep brain stimulation has evolved into an effective therapeutic option for patients with advanced Parkinson's disease. The long-term benefit of this therapeutic approach has been demonstrated in numerous studies. The cardinal motor symptoms are suppressed most effectively when the subthalamic nucleus is stimulated [4,39-53].

While successful suppression of cardinal motor symptoms is well established, only little data is available on the limitations of DBS. Although DBS is supposed to be as effective in elderly patients as in younger ones, systematic studies on the complication rate, the effectiveness and therefore the risk-benefit ratio of DBS in elderly patients are still lacking.

The two age groups investigated here were comparable with regard to their baseline clinical status prior to DBS. Both groups showed comparable improvement of the motor subscale of the UPDRS (part III) and this improvement was seen throughout the follow-up period of 24 months.

As DBS has become a routine therapeutic option, many centers now strive to develop a quality standard by establishing uniform techniques of target localization and electrode implantation. So far, no direct correlation has been established between the generous use of imaging techniques with microelectrode recording and a patient's outcome. Nevertheless, state-of-the-art imaging techniques for target definition (MRI, image fusion) are more and more replacing the older methods such as ventriculography [4,54-61]. The long-term results achieved by the two participating centers did not differ although they used different techniques for defining and locating the targets of electrode implantation (center A ventriculography and stereotactic CT, center B stereotactic CT an image fusion with contrast enhanced MPRAGE), suggesting that the technique of target localization has no significant effect on the results of DBS in our study population. Improvement of motoric symptoms confirmed the efficacy of the procedure in both groups. The rate of improvement corresponds to the results of larger multicenter trials [62-64]. The medication doses could be reduced to the same extent in both age groups. The dose reductions were in the range reported in the literature [65-68].

A uniform definition of complications does not exist. This is why complication rates reported in the literature vary widely. Many studies did not report both mechanical complications and psychic abnormalities associated with STN stimulation. Those studies that report mechanical complications provide only incomplete data on neurologic and psychic changes. Even the multicenter studies published so far present a very heterogeneous picture [28,69-77]. Only the incidences of intracerebral bleeding as the most severe complication and of infection as the most common complication are reported by all investigators.

In the present study, we defined complications as all events that prolonged a patient's hospital stay or/and caused significant morbidity. The incidence of dementia during the 2-year follow-up does not differ between the two age groups and is the same as for the natural history of Parkinson's disease. Mental alterations were frequent after bilateral STN stimulation in both age groups (Tab. 2). These complaints were independent of stimulation. There were retrospectively more related to withdrawal of medication and operative stress. Further prospective evaluation was started to systematically analyze these symptoms. Reports in the literature again present a fairly heterogeneous picture. Major differences existed between both age groups with regard to the complications that occurred: Infections were significantly more frequent in the older age group than in the younger patients (p < 0.05). A total of 7 patients died during the 2-year follow-up period. In 6 patients deaths were unrelated to surgery (2 pneumonia, 1 suspected pulmonary embolism, 3 patients with cardiac failure, all deaths >6 months postoperatively). One suicide was determined to be related to surgery. This particular patient suffered from a young onset tremordominant Parkinson's disease and has had no significant history of psychiatric disorders. After surgery he developed transient manic-depressive state, which were stimulation dependend, i.e. especially stimulation of the lower two contacts led to a worsening of manic symptoms. Although motoric improvement was significant under stimulation, psychic deteriorations limited the outcome of the patient. With maximum stimulation of 1.5 V, 90 μs and 130 Hz, tremor was only partially influenced, however without psychic symptoms. These symptoms led also to a worsening of his however previously disturbed social interactions of the patient. He got finally divorced and he committed suicide 15 months after surgery. Not counting the suicide, significantly more elderly patients died (p < 0.05) compared with the younger age group. This is not surprising if one takes into account natural life expectancy. However, as shown by the results presented here, the effectiveness of DBS is independent of patient age. This is a supporting argument against an age limit for DBS. Nevertheless, DBS should be contemplated as a therapeutic option already in younger patients and in patients with earlier stages of Parkinson's disease, for example, at the time when complications of long-term levodopa therapy first manifest themselves. With such an approach, patients can benefit from STN stimulation for a much longer period of time.

Prospective studies including a long-term follow-up of STN DBS in young-onset Parkinson patients are being prepared and will provide further evidence.

## Conclusion

Bilateral DBS for Parkinson's disease is as effective in elderly patients as it is younger individuals. Long-term observation identified no differences in the effect of DBS on cardinal symptoms. Nevertheless, DBS should be considered in patients with early stages of disease as the incidence of general complications increases with age while natural life expectancy decreases. Most mechanical complications can be avoided by using a standardized operative technique.

## Competing interests

The author(s) declare that they have no competing interests.

## Authors' contributions

JV carried out the initiiation of the study and publication and literature search for the manuscript. SH was responsible for data acquisition, carried out the statistics. CO carried out the manuscript drafting. GN participated in the design of the study and coordination. All authors read and approved the final manuscript.

## Pre-publication history

The pre-publication history for this paper can be accessed here:



## Figures and Tables

**Table 1:** Mean values of UPDRS motor scores (part III) in all patients before surgery (with/without medication) and at postoperative follow-up after 6, 12, and 24 months: significant differences between preoperative med on vs. med off in all groups

	Preoperative		12 months post				24 months post			
	med on	med off	stim on med on	stim on med off	stim off med on	stim off med off	stim on med on	stim on med off	stim off med on	stim off med off

total	30*,^#^	50**,^#^	20*	28**	40	50	26	25**	44	54
										
<65	31*,^#^	51**,^#^	21*	31**	38	50	27	21**	43	53
>65	30*,^#^	49**,^#^	18*	25**	42	49	24	30**	48	56

(^#^p < 0.05, Dunn's method), med on vs. 12 m stim on/med on (*p < 0.05, Dunn's method), (24 m n.s.), preoperative med off vs. 12 m and 24 m stim on/med off (**p < 0.05, Dunn's method), no significant differences among the age group's and total of patient's

**Table 2:** Complications in groups I and II

**Total**	**Group I <65 y (n = 37)**	**Group II >65 y (n = 36)**
Hemorrhage	0	1
Infections (generator)	1	4
Technique	1	2
Mental changes, transient	7	4
Mental changes, persistent	1	2
Death within 2 years	2	5
General (pneumonia)	2	2

total (patients)	n = 12 (32%)	n = 15 (42%)

only one death in group I was considered a complication (suicide 1 year after surgery)

**Table 3:** Stimulation Parameters

*Stimulation parameter*	*postoperatively*	*after 12 months*
• Amplitude [V]	2.3 ± 0.9	3.0 ± 0.8
• Pulse width [μsec]	88.3 ± 45.6	85.7 ± 38.7
• Frequency [Hz]	133.1 ± 7.8	135.6 ± 12.4
